# Synthetic hydrogel supports the function and regeneration of artificial ovarian tissue in mice

**DOI:** 10.1038/npjregenmed.2016.10

**Published:** 2016-07-07

**Authors:** Jiwon Kim, Amanda S Perez, Jake Claflin, Anu David, Hong Zhou, Ariella Shikanov

**Affiliations:** 1 Department of Macromolecular Science & Engineering, University of Michigan, Ann Arbor, MI, USA; 2 Department of Biomedical Engineering, University of Michigan, Ann Arbor, MI, USA

## Abstract

Many prepubertal girls and young women suffer from premature ovarian insufficiency induced by chemotherapy given for treatment of cancer and autoimmune diseases. Autotransplantation of cryopreserved ovarian tissue could restore the lost ovarian endocrine function and fertility. Unfortunately, tissue ischemia, inconsistent graft quality and the risk of reintroducing malignant cells may stand in the way of the clinical translation of this approach. To address these risks and limitations, we engineered an artificial ovarian tissue from immature follicles using a synthetic hydrogel, poly(ethylene glycol) vinyl sulfone (PEG-VS), as a supportive matrix. Enzymatically isolated follicles from 6–7-day-old mice ovaries were encapsulated in 7% PEG-VS hydrogels modified with 0.5 mmol/l RGD and crosslinked with a trifunctional matrix metalloproteinase-sensitive peptide. PEG hydrogels with the encapsulated follicles were orthotopically implanted into ovariectomised mice to investigate whether PEG hydrogel supports folliculogenesis and steroidogenesis *in vivo*. After 30 days, grafts revealed multiple fully developed antral follicles and corpora lutea, which corresponded with regular ovulation cycles and follicle-stimulating hormone (FSH) levels. The elevated levels of FSH, caused by bilateral ovariectomy, were reversed by the implanted follicles and maintained at physiological levels for 60 days. Importantly, primordial and primary follicles still represented 60% of the follicular pool, demonstrating selective recruitment of primordial follicles into the growing pool. Functioning blood vessels in the grafts 30 and 60 days after implantation proved the capability of PEG hydrogels to undergo graft remodelling and revascularisation. Our results demonstrate that PEG hydrogels with encapsulated immature ovarian follicles successfully functioned as an artificial ovarian tissue for 60 days *in vivo*.

## Introduction

The number of cancer survivors has been growing owing to advancements in anticancer treatments, and the long-term survival rates improved >80% in most childhood malignancies.^1,2^ Yet, cytotoxic chemotherapy, radiotherapy and bone marrow transplantation treatments often cause infertility and premature ovarian failure.^3,4^ Clinically approved fertility preservation options exist only for adult patients who can produce a fully mature egg.^5^ If the anticancer treatment can be delayed, then these patients can undergo hormonal stimulation to stimulate follicle development and recover mature oocytes.^6^ The retrieved oocytes can then be cryopreserved and used later for *in vitro* fertilisation treatments. Unfortunately, the option of egg preservation is not applicable to patients who require immediate treatment or prepubescent girls, whose dormant ovaries cannot produce mature eggs.^6^ Furthermore, the cytotoxic chemotherapy affects the ability of the prepubescent girls to undergo complete and physiological puberty.^7^ This eventually leads to a variety of long-term clinical complications, such as delayed growth of bones, impaired cognitive development, cardiovascular and metabolic complications.^8^

Autotransplantation of cryopreserved ovarian tissue has shown promising results for fertility and ovarian function restoration and has resulted in 60 reported live human births, since 2004.^9^ Unlike *in vitro* maturation and fertilisation of mature eggs, transplantation of ovarian tissue does not require repeated cycles of hormone stimulation, thus reducing the risk of ovarian hyperstimulation syndrome and delay in chemotherapy treatments.^10^ Despite promising results, there is a significant loss of the follicular pool owing to tissue ischemia and it requires multiple invasive procedures to extend ovarian function because of the short life span of autografts.^11–13^ Yet, the most prohibitive limitation of the ovarian tissue autotransplantation is a potential risk for reintroducing malignant cells, especially in patients with leukemia and other blood-borne cancers.^14^ Therefore, grafting of multiple isolated follicles presents a safer alternative, because individual follicles are separated from the stromal environment before being encapsulated in a three-dimensional supportive matrix.

Ovarian tissue contains follicles at different developmental stages surrounded by stroma cells and rich vasculature. Follicles, the functional units of the ovary, are composed of a germ cell (the oocyte) and layers of somatic cells (granulosa and theca cells), which are responsible for the production and metabolism of gonadal hormones, estradiol and progesterone. At birth, the human ovary contains approximately one million of immature follicles, called primordial follicles, which have the potential to develop and produce mature oocytes capable of fertilisation. This number decreases to 300,000 follicles at puberty and continues to decline until menopause.^15^ Because of the non-regenerative nature of follicles, a sufficient number of follicles must be present in the ovary in order to sustain ovarian endocrine function.

Primordial follicles, the most immature and most abundant class of follicles, constitute the ovarian reserve, and activation of a small portion of this reserve each cycle ensures ovarian function.^15^ Activated primordial follicles enter the growing pool of preantral follicles and secrete sex hormones. A burst activation of premature follicles can shorten the longevity of the graft. A successful transplantation would require significant primordial follicle population, which can contribute to monthly cycles and provide healthy, fertilisable eggs.^6,16–18^ Several reports have investigated an approach of grafting enzymatically isolated immature follicles encapsulated in naturally derived hydrogels, such as alginate,^19^ fibrin,^20–22^ fibrin and vascular endothelial growth factor,^21,23^ for delivery of isolated follicles and demonstrated complete folliculogenesis *in vivo* showing the potential of this approach. Here for the first time, we report the application of synthetic hydrogels with defined degradation kinetics and tunable physical properties^24^ to support long-term engraftment and function of the artificial ovarian tissue.

To address the limitations of fertility preservation options available only to a small subset of patients, we investigated whether an engineered ovarian tissue supports the ovarian endocrine function in sterile mice. We constructed an artificial ovary from preantral follicles encapsulated in a tunable synthetic hydrogel, poly(ethylene glycol) vinyl-sulfone (PEG-VS), as a supportive matrix. The tunable biophysical and biochemical properties^24,25^ of PEG hydrogels successfully mimic the native complexity of the extracellular matrix. End-functionalised multiarm PEG-VS allows modification with integrin-binding peptides (such as RGD (Arg-Gly-Asp)) to allow cell–matrix interactions^26,27^ and crosslinking with protease-sensitive peptides to allow cell-driven degradation and remodelling of the matrix. PEG-based matrices support folliculogenesis *in vitro*,^28^ vascularisation of tissue constructs *in vivo*^29^ and promote matrix remodelling in bone defects.^30,31^ We hypothesised that PEG-VS hydrogels modified with RGD and crosslinked with matrix metalloproteinase-sensitive peptide can serve as a supportive matrix for an engineered artificial ovarian tissue. Matrix with matrix metalloproteinase-sensitive peptide would allow cell and follicle-driven remodelling as the abundance of collagen in the natural ovarian tissues undergoes remodelling during follicular development and ovulation.^32^ We evaluated the development of immature follicles encapsulated in PEG hydrogels and assessed the graft function after orthotopic transplantation into ovariectomised mice. Our results demonstrated for the first time that synthetic PEG hydrogels with proteolytically controlled degradation successfully supported follicle growth and promoted graft remodelling *in vivo*.

## Results

### Development of immature follicles encapsulated in PEG

**Figure 1:**
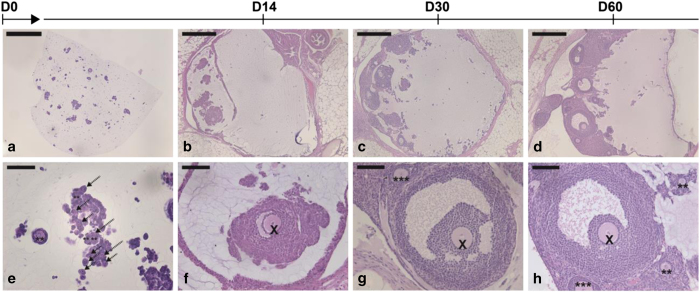
Folliculogenesis in PEG grafts. (**a**–**d**) Over 60 days, massive volumetric expansion was observed during the complete folliculogenesis, beginning with the (**e**) mixed population of primordial and primary follicles to (**f**–**h**) antral follicles, and more tissue regeneration occurred (**b**–**d**). Antrum formation was observed by day 14. By day 30 and 60, multiple antral follicles were observed. (Primordial (dotted arrow), Primary follicle (**), Secondary follicle (***), Antral follicle (X)), Scale Bar=500 μm (**a**–**d**), 50 μm (**e**), 100 μm (**f**–**h**).

**Figure 2:**
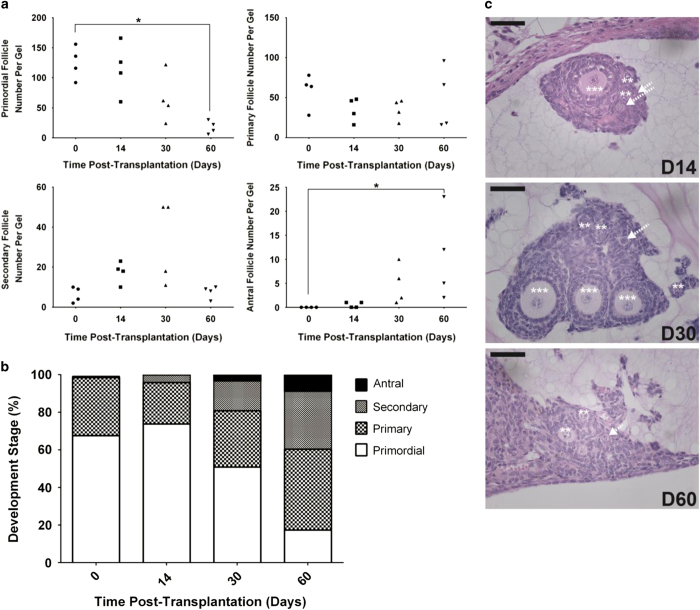
Follicle development and progression through the developmental stages. (**a**) The number of follicles in each graft at different stages after transplantation. There was a decreasing trend of the primordial follicle number in grafts over 60 days as a result of entering the growing pool every estrous cycle, ovulate and become corpus lutea. The distribution in the follicle numbers before transplantation resulted due to the loss of follicles during encapsulation process (*n*_gel_=4 from four mice, for each time point). Statistical significance was determined by the Kruskal–Wallis test followed by Dunn’s post-test (*P*<0.05). (**b**) Graft composition and percentage of the follicles at each developmental stage. After 14 days *in vivo* the majority of the implanted follicles were at primordial stage, similar to the graft composition at the implantation. After 30 days, 15% of the implanted follicles reached a preantral stage and 5% were antral. The percentage of primordial follicles decreased and the percentage of growing follicles increased over 60 days *in vivo*. 60% of immature follicular pool, including both primordial and primary follicles, was still presented in the grafts. (**c**) Primordial and primary follicles present at day 14, 30 and 60 after implantation (Primordial follicles (dashed arrow), Primary follicle (**), Secondary follicle (***)). Scale Bar=50 μm.

Histological analysis of PEG hydrogels explanted 14, 30 and 60 days after transplantation was performed to evaluate the development of the implanted follicles. The initial population of the follicles (day 0) was mostly primordial follicles (67.6%) with 30.8% primary and a small percentage (1.6%) of secondary follicles (Figure 1a,e). Antral follicles appeared 14 days after transplantation (Tx), which is consistent with the fact that the transition from primordial to antral takes 14 to 18 days during normal folliculogenesis in mice^33^ (Figure 1f). Multiple fully mature pre-ovulatory follicles were observed in the grafts retrieved after 30 (Figure 1g) and 60 days (Figure 1h) post-Tx, which indicated the progression and the extent of the follicle development during this period. From day 14 to day 60, the proportion of secondary and antral follicles increased by 7.7 times and 36 times, respectively (Figure 2b), and tissue regeneration progressed as encapsulated follicles were going through follicle development (Figure 1b–d).

### Immature follicular pool sustained

We quantified the number and the distribution of follicles per gel before and after transplantation to evaluate the follicular reserve (Figure 2a,b). There was a decreasing trend of the primordial follicle number and an increasing trend of the antral follicle number over 60 days in grafts (Figure 2a). This was a result of primordial follicles entering the growing pool every estrous cycle and reaching fully mature preovulatory follicles. The variability in the initial number of follicles encapsulated in the graft was due to the loss of follicles during the sedimentation and encapsulation processes. The expected decreasing trend of the early-stage follicle numbers in grafts (Figure 2a) indicates a functioning graft that activates a small cohort of follicles each estrous cycle. Multiple growing follicles at the primary and early secondary stages were observed 14 days after transplantation. As primordial follicles were recruited into the growing pool, a 51.4% decrease of primordial follicles occurred over 60 days *in vivo*. However, 60% of the immature follicular pool, including both primordial and primary follicles, was still present in the grafts at day 60 (Figure 2b). On the basis of histological analysis, both primordial and primary follicles were observed adjacent to secondary follicles (Figure 2c), showing selective activation and recruitment of primordial follicles into the growing pool.

### Restoration of hypothalamus–pituitary–gonadal axis

**Figure 3:**
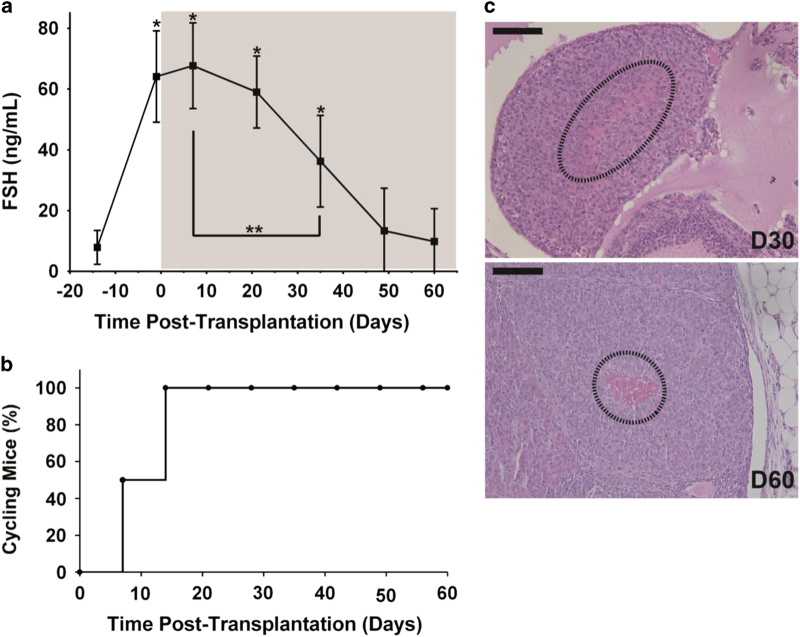
Graft function. (**a**) FSH level significantly increased post-OvX, confirming the complete OvX. For the group transplanted with PEG graft, FSH level decreased beginning at 35 days post-Tx (**), and FSH level declined to Pre-OvX levels by 49 days post-Tx, confirming the restoration of HPG axis. All the data are reported as mean±s.d. (with PEG graft: D-14–D28 (*n*_mice_=11), D35–D60 (*n*_mice_=6)). An asterisk (*) denoted significance within the group transplanted with PEG graft compared with pre-OvX (D-14). Statistical significance was determined by a one-way analysis of variance (ANOVA) followed by Tukey’s test (*P*<0.01). (**b**) Vaginal cytology demonstrated resumed cyclicity in 50% of mice after 7 days post-Tx and 100% by 14 days. Cyclicity was maintained up to 60 days, supporting 12–15 estrous cycles in mice. (*n*_mice_=4). (**c**) Corpora lutea (CL) were observed at 30 and 60 days post-Tx, in agreement with the FSH levels. CL formation suggests ovulatory events and they showed the typical characteristic of centrally located blood clot (dashed outline).

Ovariectomy (OvX), and the subsequent absence of circulating gonadal hormones produced in the ovaries, disrupts the negative-feedback loop of the hypothalamus–pituitary–gonad (HPG) axis. The most immediate and pronounced clinical outcome after removal of the ovaries is disruption and absence of regular estrous cycles as well as elevated follicle-stimulating hormone (FSH) levels. To investigate how the graft contributes to the restoration of HPG axis, FSH levels were measured before and after transplantation (Figure 3a). As expected, serum FSH levels significantly increased post-OvX (from 8 to 64 ng/ml) and the levels remained constantly elevated up to 14 days post-Tx. The elevated FSH levels, ranged from 64 to 76 ng/ml, persisted without the implantation of PEG grafts throughout the study in the control group. However, FSH levels consistently decreased in mice that received the PEG grafts, as the transplanted follicles initiated folliculogenesis and steroidogenesis. The FSH levels declined to 36 ng/ml by day 35 post-Tx, and 13 ng/ml by day 49. The physiological levels of FSH were maintained up to day 60 (10 ng/ml), confirming the restoration of the HPG axis. The resumption of the estrous cycle 14 days post-Tx in all the transplanted mice further confirmed the presence of functioning artificial ovarian tissue (Figure 3b). Presence of corpora lutea in the histological sections collected after 30 and 60 days served as an important evidence of successful ovulation, which was consistent with regular estrous cyclicity (Figure 3c).

### Graft remodelling and neovascularisation

**Figure 4:**
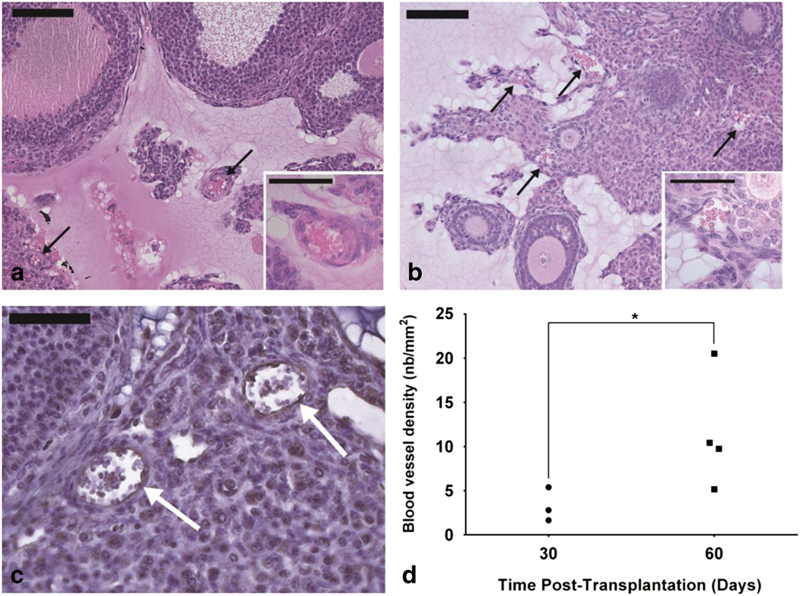
Graft remodelling and revascularisation. Functioning blood vessels (arrows) were found after (**a**) 30 days and (**b**) 60 days *in vivo*, as indicated by the presence of red blood cells (box). Scale Bar=100 μm, box: 50 μm (**c**) CD34 staining (arrows) confirmed the infiltration of blood capillaries into the graft. (**d**) Blood vessel density (number of blood vessel (nb) per mm^2^) significantly increased from 30 days to 60 days post-Tx, confirming more progressed remodelling process. Neovascularisation was observed in three out of five mice for 30 days post-Tx and four of five mice for 60 days. Statistical significance was determined by the Mann–Whitney test (*P*=0.1).

The degree of remodelling and neovascularisation of the implanted PEG hydrogel directly impacts the graft longevity. Functioning blood vessels were found after 30 (Figure 4a) and 60 (Figure 4b) days post-Tx, which was indicated by the presence of red blood cells in the lumen of the vessels. The presence of the blood capillaries was confirmed by positive CD34 immunostaining (Figure 4c), indicating PEG hydrogel’s capability of stromal cells and follicle driven remodelling. The density of blood vessels increased from 30 days post-Tx to 60 days post-Tx (Figure 4d), further indicating the continuous remodelling.

## Discussion

In this study, we engineered a synthetic PEG hydrogel that supports implantation of isolated ovarian follicles and promotes ovarian function *in vivo*. Ovarian tissue from a blood-borne cancer patient potentially could contain cancer cells and autotransplantation may lead to cancer recurrence. Our approach mitigates this problem because follicles are purified from the rest of the ovarian tissue by enzymatic tissue digestion. Several groups demonstrated that enzymatically isolated follicles could be sorted from the rest of the ovarian tissue resided with cancer cells using microfluidic device^34^ or by sedimentation.^21^ However, the survival and the function of enzymatically collected primordial follicles are low due to the lack of a structural support and cell signalling from the surrounding matrix.^35,36^ Therefore, we employed PEG hydrogels with the storage modulus of 5.2 kPa, which mimicked the stiffness of soft stromal tissues (Supplementary Figure S2). And here we demonstrated that PEG hydrogels can provide the physical support to preserve oocyte–somatic cell connections and the overall architecture of encapsulated follicles while enabling follicular expansion through degradation.

Volumetric expansion of the follicle is one of the key structural events during folliculogenesis. Starting at the primordial stage, the volumetric expansion of the growing follicle is ~300-folds in mice and about 10^5^-fold in humans when the follicle reaches antral preovulatory stages.^15,28^ As a result, the surrounding matrix of the encapsulated follicles must accommodate the volumetric expansion associated with the follicular growth, either through elastic expansion or degradation. Natural hydrogels, such as alginate and fibrin, provided an important platform to study folliculogenesis *in vitro* and *in vivo*. For example, alginate hydrogels support follicle growth and three-dimensional culture of rodent follicles. Yet, the non-degradable nature of the alginate hydrogel was prohibitive in supporting the complete development of a human follicle, which typically reaches a diameter of 20 mm at the final stages.^37^ On the other hand, the fast degrading fibrin gels provided the required physical support for the remodelling and growth of the implanted follicles, but for a limited time.^20^ These examples emphasised the importance of material properties for the follicle growth and maturation. The synthetic tunable PEG hydrogels were able to accommodate massive volumetric expansions of growing follicles within the graft as indicated by the presence of multiple fully gown antral follicles.

We also observed the development of early-stage follicles into antral follicles and corpora lutea, concluding the final stage of folliculogenesis in PEG hydrogels after 30 days *in vivo*. The proportion of growing follicles continuously increased over the 60 days post implantation, while the total number of the follicles in the grafts decreased over the same period. The natural decline in follicle numbers occurs as immature follicles enter the growing pool every estrous cycle, then mature and ovulate. Maintaining high percentage of primordial and primary follicles after 60 days *in vivo* suggested a selective activation, rather than a burst activation and premature depletion of primordial follicles, which is believed to be a common cause of an early graft failure.^11–13^ This resembles behaviour of the normal ovarian reserve where the majority of primordial follicles remain in quiescent state and only a small cohort activates each cycle.^37^

Overall, multiple fully grown antral follicles and the corpora lutea corresponded with the physiological levels of FSH. We measured the FSH levels to evaluate the function of the artificial ovarian tissue and the restoration of the endocrine HPG axis. After the complete development of the early-stage follicles, granulosa cells of antral follicles produced high levels of estrogen, resulting in the restoration of feedback to reduce FSH secretion by the pituitary gland. We demonstrated that resumed cyclicity at 14 days, and significant decrease in FSH levels at 30 days post-Tx, which corresponded with the appearance of antral follicles in the histological sections. As the proportion of growing follicles continuously increased, FSH levels further declined reaching physiological levels, confirming the restoration of the HPG axis. Regular estrous cycles were maintained up to 60 days, which is another indication of potential restoration of normal endocrine function.

The described ovarian PEG graft initially sustained follicle survival by diffusion of essential nutrients without active vasculature.^38^ However, vascularisation of the graft is needed to prevent hypoxia in the long-term, control follicular quiescence and inhibit atresia for a larger construct with multiple growing follicles.^39^ Human ovarian cortical strips are dense and have longer revascularisation periods, which negatively affect the outcomes of ovarian grafts.^12,13^ Similarly, within plasma clots, oocytes were lost by extrusion and necrosis, and more losses occurred after transplantation due to hypoxia before the graft was vascularised.^40^ The presence of functioning blood vessels containing red blood cells shows a promising result of cell-driven remodelling and tissue regeneration within PEG hydrogels. The formation of a vascular network occurred mostly around follicles due to the presence of high concentration of proangiogenic growth factors, such as vascular endothelial growth factor.^41^ The presence of stromal cells is also likely to have contributed to vascularisation process after grafting by regulating vascular remodelling and producing angiogenic factors.^42^ Neovascularisation driven by endothelial and stromal cells of the host is a promising approach to reconstructing an artificial organ for long-term applications. These results demonstrate the promising role of synthetic PEG hydrogels as a platform for reproductive tissue engineering and regenerative medicine.

## Materials and methods

### Ovariectomy and orthotopic transplantation

**Figure 5:**
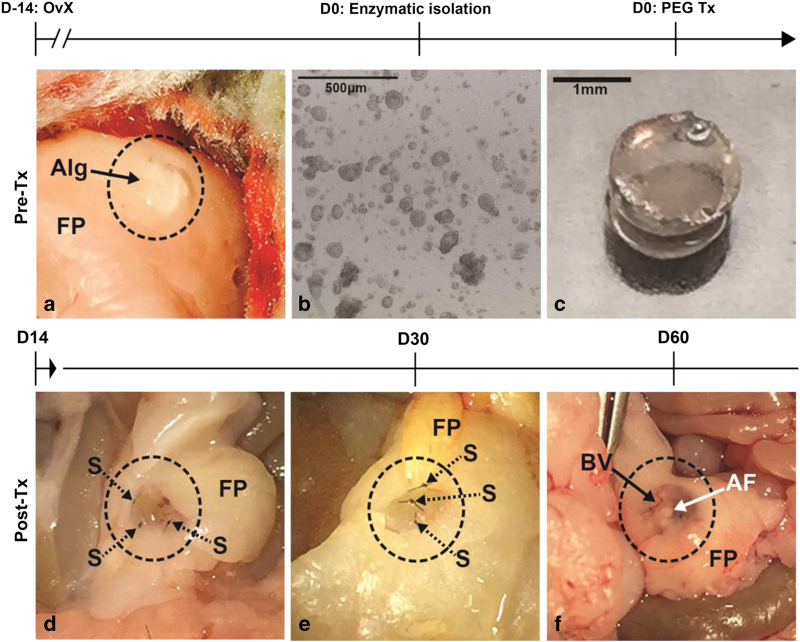
The timeline for inducing premature ovarian failure and grafting artificial ovarian tissue: (**a**) a bilateral ovariectomy (OvX) was performed to induce premature ovarian failure in mice. In order to maintain the space within the bursa after OvX, inert alginate beads (black arrow) were inserted to prevent the collapse and adhesion. (**b**) The early-stage follicles were enzymatically isolated from 6- or 7-day-old female mice. (**c**) Follicles were encapsulated in PEG hydrogels. One PEG grafts with enzymatically isolated follicles was transplanted (Tx) into each ovarian bursa, two implants per mouse. PEG grafts were retrieved (**d**) 14 (*n*_mice_=11), (**e**) 30 (*n*_mice_=6) and (**f**) 60 (*n*_mice_=6) days after Tx (FP, fat pad; S, sutures (dotted arrows); BV, blood vessels; AF, antral follicle).

A bilateral OvX was performed on 12–16-week-old mice (B6CBAF1) to mimic premature ovarian failure and absence of ovarian endocrine function. Animals were treated in agreement with the NIH Guide for the Care and Use of Laboratory Animals and the Institutional Animal Care and Use Committee (IACUC) protocol at the University of Michigan. All the surgical procedures were approved and performed according to the IACUC protocol (PRO00006459). Ovaries were removed from the ovarian bursa as previously described^43^ with some modification. Briefly, a 1.5-cm longitudinal midline incision was made in the abdominal wall using aseptic techniques and procedures. Then intraperitoneal space was exposed with an abdomen retractor. Before the ovary was removed, the ovarian blood vessels were tied to prevent bleeding after ovariectomy. To prevent the bursal cavity from collapsing, a 15-μl alginate (1.0% w/v) bead was placed inside the bursal cavity and closed with 10-0 non-absorbable sutures (Figure 5a). The same procedure was repeated for each ovary. The abdominal muscles and skin were closed with 5-0 absorbable sutures. Following recovery, the animals were housed in the animal facility for at least 14 days. Mice received analgesics for at least 48 h after surgery or as needed. Fourteen to 21 days after ovariectomy, PEG hydrogels were transplanted into both orthotopic sites as described above, following removal of the alginate bead (Figure 5d–f). Ovariectomised mice were randomly assigned to a control group that did not receive PEG grafts post-OvX. The graft function was assessed through daily vaginal cytology, serum levels of FSH and histology of the retrieved PEG-VS hydrogels after 14 (*n*_mice_=11), 30 (*n*_mice_=6) and 60 days (*n*_mice_=6) after transplantation. Power analysis was performed using Graphpad StatMate (GraphPad Prism Software, La Jolla, CA, USA) (*α*=0.05, power>80%) to determine the number of animals.

### Enzymatic follicle isolation and viability assessment

For all experiments, ovaries were isolated from 6–7-day-old female mice (B6CBAF1), separated from the connective tissues and enzymatically digested in 50 μl (13 Wünsch units per ml) Liberase DH (Roche, Indianapolis, IN, USA) in 500 μl L15 (Leibovitz’s) media (Sigma-Aldirch, St Louis, MO, USA) (Figure 5b). The total digestion time was 50 min at 37 °C, followed by 20 min of gentle pipetting of enzyme-digested pieces. The enzyme digestion was arrested by adding 10% fetal bovine serum and the digest was transferred to a tube for sedimentation. The sedimentation velocity of primordial and primary follicles was calculated according to a previously published report.^20^ After 15 min of sedimentation, the top half of the total media containing slower sinking arbitrary cells was removed. The rest of the media volume remaining follicular suspension was removed by centrifuging at 100*g* for 5 min. To determine any deleterious effects of the isolation procedure on the viability of enzymatically isolated follicles, double fluorescent labelling LIVE/DEAD Cell Imaging Kit (Invitrogen, Carlsbad, CA, USA) was performed according to the manufacturer’s protocol (Supplementary Figure S1).

### Encapsulation of enzymatically isolated follicles in PEG-VS hydrogels and graft preparation

The hydrogels were prepared by dissolving 8 arm PEG-Vinyl sulfone (PEG-VS, hexaglycerol, 40,000 g/mol, >99% purity, Jenkem Technology, Beijing, China) in 0.05 M HEPES Buffer at pH 7.66 at room temperature, followed by addition of integrin-binding peptide (RGD) (GenScript, Piscataway, NJ, USA). The hydrogels were formed via Michael-type addition chemistry with matrix metalloproteinase-sensitive tri-functional crosslinking peptides AcG**C***L*↓*GPA*G**C***L*↓*GPA***C**G (LGPA) (1217.45 g/mol, >90% purity, GenScript, cleavage site indicated by ↓, cysteines with reactive thiols are in bold). The stoichiometric ratio of −VS to thiol (−SH) groups was kept at 1:1 ratio for all experiments. PEG hydrogels were modified with the integrin-binding peptide G**C**GYG*RGD*SPG (RGD) (1067.10 g/mol, GenScript) to allow cell adhesion and migration. The modified PEG solution was kept at room temperature for 15 min to allow the RGD peptide to chemically bind to the PEG precursor, and 6.5 μl of the modified PEG solution was then pipetted into the concentrated follicle suspension (1 μl) and gently mixed. The LGPA crosslinker was dissolved in HEPES buffer and 5.5 μl of the crosslinking peptide solution was added to the mixture of PEG-VS and follicle suspension. Two grafts with follicles (6.5 μl each) were allowed to crosslink on a heating plate at 37 °C for 10 min to complete the Michael-type addition reaction. Upon complete gelation, these hydrogels were transplanted immediately into the ovarian bursa (Figure 5c).

### Functionality of the artificial ovarian tissue

#### Vaginal cytology

The presence of estrous cycle in the transplanted animals was determined by daily vaginal cytology. The animals were gently restrained by the tail and 0.1–0.2 ml of saline was pushed using a syringe or a pipette in and out of the vaginal opening to collect cells to identify cycle stage every day, before and after transplantation. The onset and cessation of cyclicity were determined by the cell population in the vaginal lavage.

#### Blood collection for measurements of follicle-stimulating hormone in serum

Lateral tail vein blood (0.5–1% volume of the total body weight: approximately 0.075–0.15 ml from a mouse weighing 15 g) was collected every 2 weeks to compare FSH levels pre- and post-transplantation. The mouse was restrained in a mouse trap to allow easy access to the tail. The lower one half of the tail was cleaned with 70% alcohol and a small incision in the tail vein at the distal end of the tail was made using a sharp scalpel or razor blade. A new scalpel was used for each animal. Seventy five to 150 μl blood was collected in a capillary tube and left at 4 °C overnight to allow blood to clot. Then, 10–20 μl serum was aspirated from the sample following centrifugation at 10,000 r.p.m. at 4 °C for 10 min and stored at −20 °C. At the terminal time point of the experiment, the blood was collected via cardiac puncture. All samples were labelled numerically to blind any correlation to time points or group assignments. The FSH levels were measured with Radioimmunoassay at Ligand Assay and Analysis Core Facility at University of Virginia Center for Research in Reproduction.

### Histological tissue analysis

After 14 (*n*_mice_=11), 30 (*n*_mice_=6) and 60 days (*n*_mice_=6) following transplantation, PEG-VS hydrogels were retrieved and fixed in Bouin’s fixative solution (Sigma-Aldrich) overnight, and then transferred to 70% EtOH at 4 °C until processing. The number of follicles and their distribution on day 0 (pretransplantation) was characterised using HistoGel (Richard-Allan Scientific, San Diego, CA, USA) using a similar procedure for encapsulation in PEG. After fixation, all samples were processed at the Histology Core in Microscopy & Image Analysis Laboratory at the University of Michigan. Samples were embedded in paraffin and serially sectioned at a 7-μm thickness and were stained with haematoxylin and eosin to identify and count follicles at all the developmental stages. Primordial follicles were identified by the nucleus, which is surrounded by a single layer of flattened squamous follicular cells. A primary follicle was defined as an oocyte surrounded by a single layer of cuboidal granulosa cells. A secondary follicle has two or more layers of cuboidal granulosa cells, but no antrum, and the presence of a fluid-filled antrum defined an antral follicle. Extensive permeation of red blood cells into the ovulated follicle and luteinised granulosa cells were defined as corpora lutea.

Identified follicles were quantified by counting every-other section of every-other slide (four sections per slide) to prevent counting a follicle more than once.^20^ As every-other slide was counted, a correction factor was applied to adjust for the counting rules. The oocyte diameter for primordial and primary follicles averaged 14 μm,^44^ meaning the same follicle would appear only on two consecutive sections. Therefore, the numbers of primordial and primary follicles were doubled since every-other slides was counted. Primary, secondary and antral follicles were counted when the nucleolus of the oocyte was present. This was done to prevent double counting of large growing follicles (secondary and antral) and undercounting smaller follicles.

### Immunohistochemistry

To analyse the neovascularisation within transplanted PEG hydrogels, paraffin-sectioned slides were stained for mouse endothelial cells. First, sections were deparaffinised with Xylene and rehydrated in 100% ethanol. Slides were incubated in 3% H_2_O_2_ for 30 min at room temperature to block any endogenous peroxidase activity. Then, slides were incubated in 10 mmol/l Tris EDTA (pH 9) solution for 20 min at 96 °C and additional 20 min at room temperature for antigen retrieval. Afterwards, non-specific bindings were blocked by the protein block (Abcam, Cambridge, UK) for 1 h followed by overnight incubation at 4 °C with primary antibodies—rabbit anti-mouse CD34 antibody (1:500 dilution; Abcam, catalog#ab81289) dissolved in Tris-buffered saline with 1% Normal Rabbit Serum (Sigma-Aldrich) and 0.1% bovine serum albumin (Fisher Scientific, Waltham, MA, USA). Following incubation overnight, slides were treated for 20 min each with the biotinylated goat anti-polyvalent and streptavidin peroxidase, provided in Rabbit Specific HRP/DAP Detection IHC kit (Abcam). Haematoxylin was used for counterstaining. Healthy mouse uterus was used as positive-control and negative-control slides were incubated without the presence of primary antibody (Supplementary Figure S3). The identical procedure was followed for both positive and negative control as described.

### Statistical analysis

All statistical analyses were performed using GraphPad Prism (GraphPad Prism) and power analyses were performed using Graphpad StatMate (GraphPad Prism Software; *α*=0.05, power >80%). Homogeneity of variance was determined with an F-test. For sample sizes (*n*>5), data are reported as mean±s.d. of measurements, and statistical analyses were performed with one-way analysis of variance followed by Tukey’s post-test. And statistical significance was set at *P*<0.05. For sample sizes (*n*<5), individual data points were plotted and the statistical analyses were performed with the Kruskal–Wallis test followed by Dunn’s post-test (Figure 2a), or Mann–Whitney test (Figure 4d).

## References

[bib1] Resetkova, N., Hayashi, M., Kolp, L. A. & Christianson, M. S. Fertility preservation for prepubertal girls: update and current challenges. Curr. Obstet. Gynecol. Rep. 2, 218–225 (2013). 25110617 10.1007/s13669-013-0060-9 PMC4125124

[bib2] Smith, M. A., Altekruse, S. F., Adamson, P. C., Reaman, G. H. & Seibel, N. L. Declining childhood and adolescent cancer mortality. Cancer 120, 2497–2506 (2014). 24853691 10.1002/cncr.28748 PMC4136455

[bib3] Meirow, D. & Nugent, D. The effects of radiotherapy and chemotherapy on female reproduction. Hum. Reprod. Update 7, 535–543 (2001). 11727861 10.1093/humupd/7.6.535

[bib4] Green, D. M. et al. Ovarian failure and reproductive outcomes after childhood cancer treatment: Results from the childhood cancer survivor study. J. Clin. Oncol. 27, 2374–2381 (2009). 19364956 10.1200/JCO.2008.21.1839 PMC2677923

[bib5] Ethics Committee of American Society for Reproductive Medicine. Fertility preservation and reproduction in patients facing gonadotoxic therapies: a committee opinion. Fertil. Steril. 100, 1224–1231 (2013). 24094423 10.1016/j.fertnstert.2013.08.041

[bib6] Jeruss, J. S. & Woodruff, T. K. Preservation of fertility in patients with cancer. N. Engl. J. Med. 360, 902–911 (2009). 19246362 10.1056/NEJMra0801454 PMC2927217

[bib7] Diller, L. Clinical practice. adult primary care after childhood acute lymphoblastic leukemia. N. Engl. J. Med. 365, 1417–1424 (2011). 21995389 10.1056/NEJMcp1103645

[bib8] Shuster, L. T., Rhodes, D. J., Gostout, B. S., Grossardt, B. R. & Rocca, W. A. Premature menopause or early menopause: long-term health consequences. Maturitas 65, 161–166 (2010). 19733988 10.1016/j.maturitas.2009.08.003 PMC2815011

[bib9] Donnez, J. & Dolmans, M. M. Ovarian cortex transplantation: 60 reported live births brings the success and worldwide expansion of the technique towards routine clinical practice. J. Assist. Reprod. Genet. 32, 1167–1170 (2015). 26210678 10.1007/s10815-015-0544-9 PMC4554373

[bib10] Fauser, B. C. et al. Minimal ovarian stimulation for IVF: appraisal of potential benefits and drawbacks. Hum. Reprod. 14, 2681–2686 (1999). 10548600 10.1093/humrep/14.11.2681

[bib11] Donnez, J. et al. Restoration of ovarian activity and pregnancy after transplantation of cryopreserved ovarian tissue: a review of 60 cases of reimplantation. Fertil. Steril. 99, 1503–1513 (2013). 23635349 10.1016/j.fertnstert.2013.03.030

[bib12] Kim, S. S., Soules, M. R. & Battaglia, D. E. Follicular development, ovulation, and corpus luteum formation in cryopreserved human ovarian tissue after xenotransplantation. Fertil. Steril. 78, 77–82 (2002). 12095494 10.1016/s0015-0282(02)03144-8

[bib13] Donnez, J., Squifflet, J. & Dolmans, M. M. Frozen-thawed ovarian tissue retransplants. Semin. Reprod. Med. 27, 472–478 (2009). 19806516 10.1055/s-0029-1241057

[bib14] Dolmans, M. M., Luyckx, V., Donnez, J., Andersen, C. Y. & Greve, T. Risk of transferring malignant cells with transplanted frozen-thawed ovarian tissue. Fertil. Steril. 99, 1514–1522 (2013). 23541406 10.1016/j.fertnstert.2013.03.027

[bib15] Picton, H. M. Activation of follicle development: the primordial follicle. Theriogenology 55, 1193–1210 (2001). 11327680 10.1016/s0093-691x(01)00478-2

[bib16] Smitz, J. et al. Current achievements and future research directions in ovarian tissue culture, in vitro follicle development and transplantation: Implications for fertility preservation. Hum. Reprod. Update 16, 395–414 (2010). 20124287 10.1093/humupd/dmp056 PMC2880913

[bib17] Nieman, C. L. et al. Cancer survivors and infertility: A review of a new problem and novel answers. J. Support. Oncol. 4, 171–178 (2006). 16669459

[bib18] West, E. R. et al. Preserving female fertility following cancer treatment: current options and future possibilities. Pediatr. Blood Cancer 53, 289–295 (2009). 19301373 10.1002/pbc.21999 PMC3081672

[bib19] Vanacker, J., Dolmans, M. M., Luyckx, V., Donnez, J. & Amorim, C. A. First transplantation of isolated murine follicles in alginate. Regen. Med. 9, 609–619 (2014). 25372078 10.2217/rme.14.33

[bib20] Smith, R. M. et al. Fibrin-mediated delivery of an ovarian follicle pool in a mouse model of infertility. Tissue Eng. Part A 20, 3021–3030 (2014). 24802617 10.1089/ten.tea.2013.0675 PMC4229702

[bib21] Kniazeva, E. et al. Primordial follicle transplantation within designer biomaterial grafts produce live births in a mouse infertility model. Sci. Rep. 5, 17709 (2015). 26633657 10.1038/srep17709 PMC4668556

[bib22] Chiti, M. C. et al. Influence of follicle stage on artificial ovary outcome using fibrin as a matrix. Hum. Reprod. 31, 427–435 (2016). 26628641 10.1093/humrep/dev299

[bib23] Shikanov, A. et al. Fibrin encapsulation and vascular endothelial growth factor delivery promotes ovarian graft survival in mice. Tissue Eng. Part A 17, 3095–3104 (2011). 21740332 10.1089/ten.tea.2011.0204 PMC3226061

[bib24] Kim, J. et al. Characterization of the crosslinking kinetics of multi-arm poly(ethylene glycol) hydrogels formed via michael-type addition. Soft Matter 12, 2076–2085 (2016). 26750719 10.1039/c5sm02668g PMC4749500

[bib25] Lutolf, M. P. & Hubbell, J. A. Synthesis and physicochemical characterization of end-linked poly(ethylene glycol)-co-peptide hydrogels formed by michael-type addition. Biomacromolecules 4, 713–722 (2003). 12741789 10.1021/bm025744e

[bib26] Hern, D. L. & Hubbell, J. A. Incorporation of adhesion peptides into nonadhesive hydrogels useful for tissue resurfacing. J. Biomed. Mater. Res. 39, 266–276 (1998). 9457557 10.1002/(sici)1097-4636(199802)39:2<266::aid-jbm14>3.0.co;2-b

[bib27] Howe, A., Aplin, A. E., Alahari, S. K. & Juliano, R. L. Integrin signaling and cell growth control. Curr. Opin. Cell Biol. 10, 220–231 (1998). 9561846 10.1016/s0955-0674(98)80144-0

[bib28] Shikanov, A., Smith, R. M., Xu, M., Woodruff, T. K. & Shea, L. D. Hydrogel network design using multifunctional macromers to coordinate tissue maturation in ovarian follicle culture. Biomaterials 32, 2524–2531 (2011). 21247629 10.1016/j.biomaterials.2010.12.027 PMC3040241

[bib29] Vigen, M., Ceccarelli, J. & Putnam, A. J. Protease-sensitive PEG hydrogels regulate vascularization in vitro and in vivo. Macromol. Biosci. 14, 1368–1379 (2014). 24943402 10.1002/mabi.201400161 PMC4198447

[bib30] Pratt, A. B., Weber, F. E., Schmoekel, H. G., Muller, R. & Hubbell, J. A. Synthetic extracellular matrices for in situ tissue engineering. Biotechnol. Bioeng. 86, 27–36 (2004). 15007838 10.1002/bit.10897

[bib31] Lutolf, M. P. et al. Repair of bone defects using synthetic mimetics of collagenous extracellular matrices. Nat. Biotechnol. 21, 513–518 (2003). 12704396 10.1038/nbt818

[bib32] Ny, T., Wahlberg, P. & Brandstrom, I. J. Matrix remodeling in the ovary: regulation and functional role of the plasminogen activator and matrix metalloproteinase systems. Mol. Cell Endocrinol. 187, 29–38 (2002). 11988309 10.1016/s0303-7207(01)00711-0

[bib33] McGee, E. A. & Hsueh, A. J. Initial and cyclic recruitment of ovarian follicles. Endocr. Rev. 21, 200–214 (2000). 10782364 10.1210/edrv.21.2.0394

[bib34] Lai, D. et al. Simple multi-level micochannel fabrication by pseudo-grayscale backside diffused light lithography. RSC Adv. 3, 19467–19473 (2013). 24976950 10.1039/C3RA43834A PMC4066982

[bib35] Hovatta, O., Wright, C., Krausz, T., Hardy, K. & Winston, R. M. Human primordial, primary and secondary ovarian follicles in long-term culture: Effect of partial isolation. Hum. Reprod. 14, 2519–2524 (1999). 10527981 10.1093/humrep/14.10.2519

[bib36] Abir, R. et al. Morphological study of fully and partially isolated early human follicles. Fertil. Steril. 75, 141–146 (2001). 11163829 10.1016/s0015-0282(00)01668-x

[bib37] Gougeon, A. Regulation of ovarian follicular development in primates: Facts and hypotheses. Endocr. Rev. 17, 121–155 (1996). 8706629 10.1210/edrv-17-2-121

[bib38] Griffith, C. K. et al. Diffusion limits of an in vitro thick prevascularized tissue. Tissue Eng. 11, 257–266 (2005). 15738680 10.1089/ten.2005.11.257

[bib39] Fortune, J. E., Cushman, R. A., Wahl, C. M. & Kito, S. The primordial to primary follicle transition. Mol. Cell Endocrinol. 163, 53–60 (2000). 10963874 10.1016/s0303-7207(99)00240-3

[bib40] Gosden, R. G. Restitution of fertility in sterilized mice by transferring primordial ovarian follicles. Hum. Reprod. 5, 499–504 (1990). 2394782 10.1093/oxfordjournals.humrep.a137132

[bib41] Geva, E. & Jaffe, R. B. Role of vascular endothelial growth factor in ovarian physiology and pathology. Fertil. Steril. 74, 429–438 (2000). 10973633 10.1016/s0015-0282(00)00670-1

[bib42] Dissen, G. A., Lara, H. E., Fahrenbach, W. H., Costa, M. E. & Ojeda, S. R. Immature rat ovaries become revascularized rapidly after autotransplantation and show a gonadotropin-dependent increase in angiogenic factor gene expression. Endocrinology 134, 1146–1154 (1994). 8119153 10.1210/endo.134.3.8119153

[bib43] Liu, L. et al. Restoration of fertility by orthotopic transplantation of frozen adult mouse ovaries. Hum. Reprod. 23, 122–128 (2008). 17993475 10.1093/humrep/dem348

[bib44] Bristol-Gould, S. K. et al. Postnatal regulation of germ cells by activin: The establishment of the initial follicle pool. Dev Biol. 298, 132–148 (2006). 16930587 10.1016/j.ydbio.2006.06.025

