# Supplementary material for: Synthetic hydrogel supports the function and regeneration of artificial ovarian tissue in mice
Source: NPJ Regen Med. 2016 Jul 7;1:16010–. doi: 10.1038/npjregenmed.2016.10 (PMC5573242; doi:10.1038/npjregenmed.2016.10)

**Supplementary figures**

***Figure S1-*** LIVE/DEAD staining of enzymatically isolated follicles: To determine deleterious effects of isolation procedure on the viability of enzymatically isolated follicle **(A)**, double fluorescent labeling LIVE/DEAD Cell Imaging Kit (Invitrogen) was performed. The enzymatically isolated follicle’s viability was confirmed by high Calcien AM (Green) staining **(B)**.

**
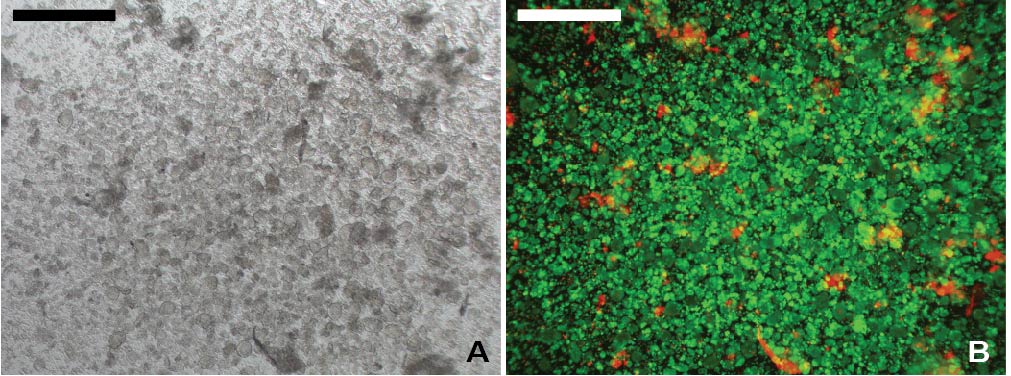
**

***Figure S2*** – Storage modulus (G’) of 7% PEG-8VS (0.5mM RGD) crosslinked with LGPA: Frequency sweep test was performed at 10% strain to measure the storage modulus (G’) of 7% PEG-8VS (0.5mM RGD) crosslinked with LGPA using AR-G2 rheometer (TA Instruments). These hydrogels were swollen in miliQ water for 24 hours before the measurement.


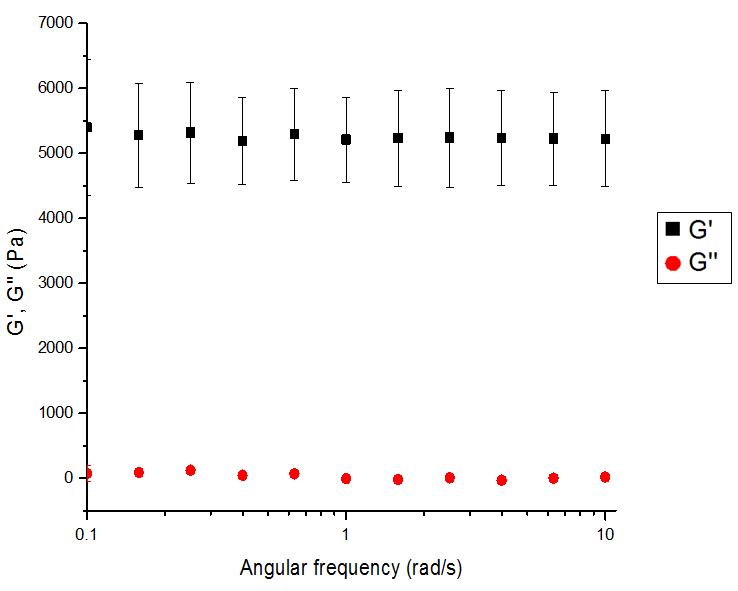


***Figure S3 -*** Healthy mouse uterus was used as positive control **(A)** and negative control **(B)** slides were incubated without the presence of primary antibody. Positive CD34 staining was only observed with the presence of primary antibody, confirming its specificity. Scale bar: 50 µm.


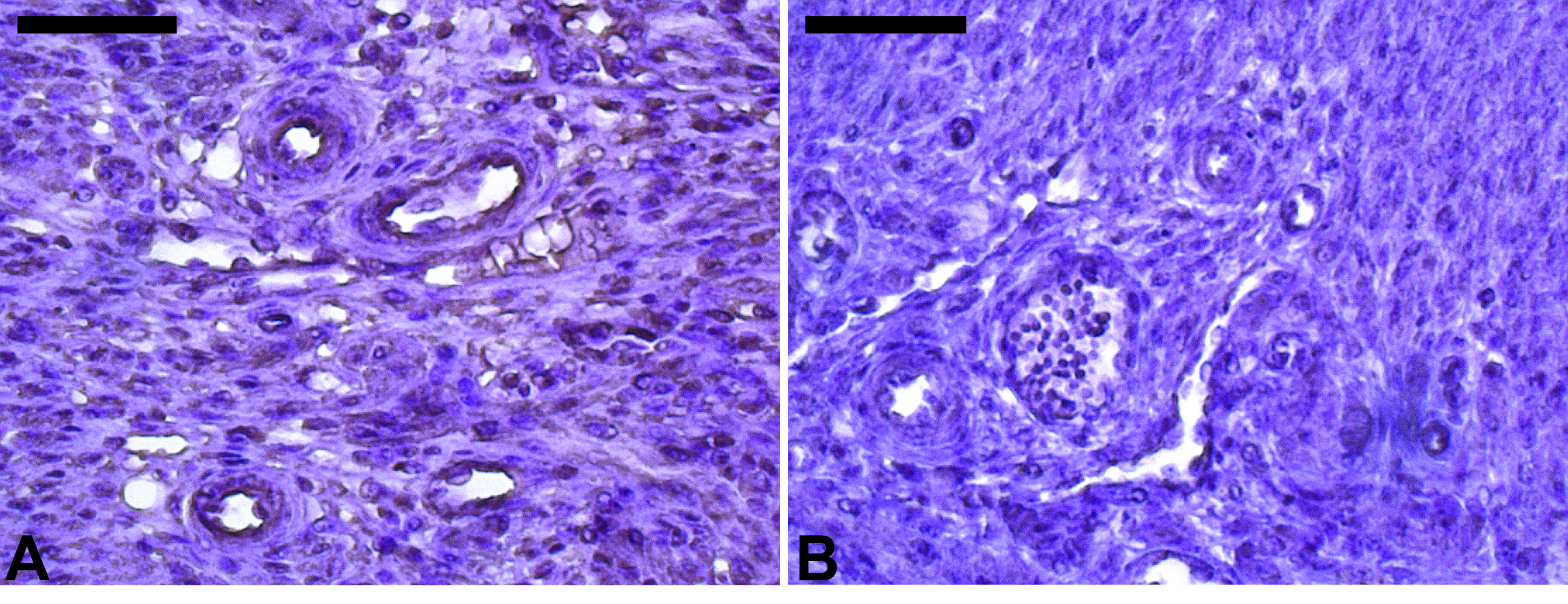

Supplement: Supplementary Figures [file npjregenmed201610-s1.doc]
